# Could wastewater-based surveillance be key to combating antimicrobial resistance?

**DOI:** 10.1128/mbio.02778-25

**Published:** 2025-10-13

**Authors:** José L. Balcázar

**Affiliations:** 1 Catalan Institute for Water Research (ICRA-CERCA), Girona, Spain; Johns Hopkins University, Baltimore, Maryland, USA

**Keywords:** antimicrobial resistance, wastewater-based surveillance, community level

## Abstract

Antimicrobial resistance (AMR) is one of the most urgent health threats of the 21st century. Surveillance is needed to enable timely interventions, close knowledge gaps, and anticipate long-term trends. Current frameworks rely heavily on clinical data, which often fail to capture population-level dynamics. Wastewater-based surveillance (WBS) offers a complementary approach by detecting antibiotic resistance genes (ARGs) in sewage. In AMR surveillance, early warning includes the detection of novel or clinically relevant ARGs, including those carried by mobile genetic elements (MGEs) before they affect clinical outcomes. WBS can also reveal resistome composition, dissemination routes, and ecological drivers of AMR. This is especially relevant in settings with poor sanitation, high exposure, and limited clinical reporting. Unlocking its potential will require harmonized protocols, sustained investment, and strong ethical measures. Within a One Health framework, WBS can strengthen equitable and evidence-based strategies against AMR.

## PERSPECTIVE

Antimicrobial resistance (AMR) causes an estimated 1.14 million deaths annually and could lead humanity toward a post-antibiotic era, with deaths projected to reach 1.91 million by 2050 (1). The COVID-19 pandemic demonstrated the potential of wastewater-based surveillance (WBS) to monitor viral transmission at the population level through community sewage (2). Despite its proven utility, WBS remains largely overlooked in efforts to address AMR.

Large-scale sewage genomics has mapped global resistome diversity (3), revealing hidden reservoirs of antibiotic resistance genes (ARGs) often missed by clinical surveillance. However, a clearer definition of what constitutes “resistance” for monitoring is needed, including clinically important ARGs, novel ARGs in environmental reservoirs, ARGs linked to mobile genetic elements (MGEs), and those associated with specific pathogens (4, 5). Each category has distinct methodological and epidemiological implications. Surveillance strategies should clearly define which resistance determinants are prioritized, as this decision shapes both methodology and applicability.

As the AMR crisis intensifies, driven by climate change, pollution, and inadequate sanitation, WBS provides a cost-effective tool to detect emerging trends and map dissemination pathways crucial for stewardship (6). Its value extends beyond early warning, revealing the circulating resistome as well as the ecological and social drivers of its spread. In AMR, early detection includes identifying plasmids carrying multiple ARGs before they circulate widely (7), observing known ARGs in new pathogen hosts, or detecting their appearance in regions or communities where they were absent. This perspective enables timely interventions and deepens understanding of long-term resistance dynamics, which are essential for effective policy and public health action.

## WBS CAN SUPPORT EFFORTS TO COMBAT THE GLOBAL AMR CRISIS

WBS, first applied in poliovirus detection in 1939, was widely adopted during the coronavirus disease 2019 (COVID-19) pandemic, particularly for monitoring severe acute respiratory syndrome coronavirus 2 (SARS-CoV-2) transmission at the population level (8). Advances in real-time PCR, high-throughput sequencing, and bioinformatics now enable rapid and cost-effective sewage analysis covering a broad range of biological markers. As AMR emerges as one of the most pressing global health threats, WBS provides an accessible approach that complements conventional monitoring systems. Clinical surveillance is essential but limited, particularly in low- and middle-income countries (LMICs) where reliable AMR data are scarce.

A global metagenomic survey of sewage from 79 sites in 60 countries revealed strong associations between the abundance and diversity of ARGs and socioeconomic, health, and environmental conditions, including sanitation infrastructure and access to clean water (3). Another study analyzing 757 sewage samples from 243 cities across 101 countries revealed marked regional variations in resistome profiles (9). Notably, 49 common ARGs were detected across diverse genetic environments, including plasmids, suggesting both localized circulation and potential for global dissemination. These data support targeted public health interventions and optimize resource allocation for antimicrobial stewardship.

Given this potential, WBS is especially relevant in regions where AMR is intensified by inadequate sanitation, high levels of antibiotic pollution, and limited access to healthcare infrastructure (5, 6, 10). However, implementation remains challenging in many LMICs, where non-sewered sanitation systems, including latrines, septic tanks, and open drains, complicate systematic sampling and may introduce biases (10, 11).

In addition to surveillance, WBS can actively guide local interventions. Spatiotemporal mapping of ARG abundance can identify environmental AMR hotspots, supporting targeted wastewater treatment improvements, such as upgrading disinfection processes, optimizing biological treatment to reduce ARG loads, or implementing advanced oxidation technologies, alongside hygiene promotion initiatives (12). At the hospital level, outbreaks may involve MGEs that transfer several ARGs across different pathogens in sink drains or wastewater pipes, creating hotspots of resistance that can guide prescribing and hygiene measures (13). Detecting such events could help physicians adjust empirical prescribing and alert hygiene teams to mitigate localized hotspots of resistance. Moreover, when integrated with data on pharmaceutical residues and pathogen loads, WBS enables comprehensive assessments of how antibiotic use influences the emergence and spread of AMR across diverse ecological and socioeconomic settings (14, 15).

The adaptability of WBS across spatial scales, from individual buildings to entire cities, makes it a key tool for One Health surveillance, which integrates human, animal, and environmental health (16). Monitoring wastewater from hospitals, dermatology clinics, livestock operations, aquaculture systems, and pharmaceutical manufacturing facilities alongside municipal sewage therefore offers a comprehensive view of environmental AMR pressures (5). This integrated perspective is especially relevant as ARGs circulate among human, animal, and environmental reservoirs, particularly in regions experiencing rapid urbanization and agricultural intensification (17). To fully realize this potential, advances in sequencing technologies are essential. Metagenomics provides culture-independent insights into the resistome, but challenges include high detection limits and the difficulty of assigning ARGs to hosts (3, 6, 18). Harmonization of pipelines is actively being developed (19). Short-read metagenomics continues to be valuable for large-scale mapping of resistome diversity and comparative analysis across regions, although assemblies can be incomplete and may limit the ability to resolve the genomic environment of ARGs. Long-read sequencing offers greater resolution by linking ARGs to hosts or plasmids, enhancing the study of co-selection processes and improving recovery of MGEs (19). Complementary approaches such as targeted whole-genome sequencing of priority ESKAPEE pathogens (*Enterococcus faecium*, *Staphylococcus aureus*, *Klebsiella pneumoniae*, *Acinetobacter baumannii*, *Pseudomonas aeruginosa*, *Enterobacter* spp., and *Escherichia coli*), are also essential for identifying emerging multidrug-resistant lineages. Wastewater composition is highly variable across catchments, with inputs from hospitals, farms, industrial facilities, or municipal sources, and systematic metadata collection is critical for robust interpretation (15). Building on these technological and methodological advances, wastewater-based epidemiology (WBE) has emerged as a key application of WBS data, providing insights into population-level trends (20, 21). For AMR, however, WBS also informs on ecological and genetic processes that extend beyond epidemiological estimates.

Strengthening the role of WBS in AMR surveillance will require long-term investment and international coordination to ensure standardization, data sharing, and robust ethical frameworks (21, 22). Although sequencing data are typically filtered, personal information can still appear in raw data sets, especially in small catchments. Transparent governance is essential to minimize risks of stigmatization. Along with expanding infrastructure in low-resource settings, global efforts should prioritize integrating WBS with clinical and environmental data streams to deliver actionable insights into resistance dynamics. With such support, WBS can become an integral component of One Health surveillance (23), bridging critical data gaps and reinforcing coordinated responses at both local and global levels.

## OPPORTUNITIES AND RISKS: ETHICS, EQUITY, AND INFRASTRUCTURE

Integrating WBS into AMR surveillance presents major opportunities, but also technical, ethical, and infrastructural challenges that must be addressed to ensure responsible and equitable implementation globally.

- Technical limitations remain a persistent obstacle. Despite advances in metagenomics and bioinformatics, assigning specific ARGs to their hosts or MGEs remains difficult. This reduces the accuracy of risk assessments and constrains the design of targeted interventions. Standardization of sampling strategies, including grab versus composite sampling, normalization methods, and flow measurements, is required to allow valid comparisons across regions and over time. To address this limitation, combining short- and long-read sequencing with improved bioinformatic pipelines could enhance host assignment and plasmid reconstruction, while international interlaboratory comparison studies would help validate and harmonize methods across settings.
- Ethical and privacy considerations are critical. Although sequencing data are usually filtered, personal information can still appear in raw data sets, especially when samples come from small catchments, such as prisons or hospitals. Transparent governance is required to prevent risks of stigmatization and to separate public health objectives from law enforcement. Experiences from wastewater-based opioid monitoring have shown that the misuse or misinterpretation of data can undermine public trust (24).
- Infrastructure disparities represent another barrier. While centralized wastewater systems are common in high-income countries, many LMICs rely on non-sewered sanitation systems, which limit the feasibility of conventional, sampling-based WBE approaches. This may widen the global surveillance gap. Surface water monitoring and decentralized sampling can help to track resistance in underserved regions, although feasibility and effectiveness remain debated (25, 26).
- Environmental justice and climate vulnerability add further complexity to the landscape. Communities without adequate sanitation, particularly in informal settlements or areas exposed to flooding, face disproportionate risks of ARG exposure through contaminated water. Climate-driven events, such as heatwaves and extreme floods, can accelerate horizontal gene transfer and facilitate the spread of ARGs, especially in regions with low adaptive capacity. Equity must be central to WBS frameworks, including gender-sensitive and community-based strategies.
- Data interpretation and actionability remain a major frontier. The complexity of resistome profiles, particularly in metagenomic data sets, calls for integrated analytical frameworks. Without platforms linking environmental, clinical, agricultural, and pharmaceutical data, insights from WBS risk remaining fragmented. Developing open-source, FAIR-compliant pipelines and dashboards is essential to translate results into practical interventions and improve forecasting.

## TRANSFORMATIVE PRIORITIES: FUNDING, INTEGRATION, AND POLICY FRAMEWORKS

To unlock the full potential of WBS for AMR surveillance, coordinated changes are needed in funding, institutional integration, ethical governance, and technological infrastructure.

First, funding mechanisms and institutional structures should position WBS as an integral part of AMR monitoring. This includes support from international financing programs such as the Global AMR Innovation Fund and environmental health initiatives, while also prioritizing pilot projects in low-resource settings. Demonstrating feasibility and cost-effectiveness in such settings is fundamental to support broader policy adoption and ensure long-term sustainability (27). Efforts should also include awareness and education programs to strengthen public understanding of AMR and build local capacity for sustained surveillance.

Second, national surveillance frameworks should formally incorporate WBS as a complementary source of AMR data. Integration into systems, such as the Global Antimicrobial Resistance and Use Surveillance System (GLASS), is essential (28). Establishing sentinel sites at strategically selected urban centers and high-risk facilities would enable more representative monitoring. Regular reporting of WBS-derived AMR indicators, aligned with clinical and agricultural data sets, would strengthen data triangulation and improve comparability across sectors. In developing countries, international support should prioritize the creation of locally adapted guidelines and capacity-building programs to ensure feasible and equitable implementation.

Third, robust ethical and governance mechanisms are essential. International bodies, such as the World Health Organization (WHO) and the United Nations Environment Program (UNEP), should lead the development of global guidelines that address privacy protections, consent procedures for catchment-level monitoring, data stewardship, and standards for public reporting. At the local level, culturally sensitive, community-driven consent models can help build public trust and mitigate the risk of stigmatization associated with environmental surveillance.

Fourth, international standardization is crucial to ensure the reliability and interoperability of WBS data. Clear International Organization for Standardization (ISO)-like protocols should guide sampling, processing, sequencing, and interpretation. Open-access, FAIR-compliant pipelines that integrate with resistance gene databases, such as the Comprehensive Antibiotic Resistance Database (CARD) (29) or ResFinder (30), would facilitate cross-jurisdictional data use.

Fifth, targeted investment in technological innovation is necessary to ensure the feasibility of WBS across diverse global settings. This includes supporting the development of portable, low-cost molecular diagnostic platforms, such as digital PCR, for the rapid detection of ARGs in resource-limited environments. Innovations in treatment processes are equally important, such as solar-driven advanced oxidation or tailored disinfection methods, which can reduce the environmental persistence and spread of ARGs (31).

Finally, the One Health approach, integrating human, animal, and environmental health, should guide all coordination efforts. This approach is promoted by WHO and recognized in G7 and G20 agendas, as it links AMR indicators across sectors and strengthens policy coherence. Cross-sectoral collaboration involving public health, veterinary medicine, agriculture, and environmental protection is essential for the effective interpretation and application of WBS data. Indicators derived from WBS may serve as real-time metrics to assess the impact of AMR policies, including limits on antibiotic discharge, improvements in sanitation infrastructure, and public awareness campaigns. Clear guidelines on the safe disposal of unused or excess antibiotics from hospitals, clinics, and households are also critical to limit environmental contamination and downstream selection pressures.

The emergence of initiatives such as the U.S. National Wastewater Surveillance System (NWSS) highlights the feasibility of large-scale AMR monitoring through WBS (32). However, global adoption will ultimately depend on sustained political commitment, regulatory alignment, and explicit integration of WBS into multilateral health financing agendas. Together, these priorities highlight that WBS is not only a technical innovation but also a societal and policy challenge, requiring global collaboration to translate surveillance data into effective action against AMR.

## CONCLUSIONS

WBS has evolved rapidly from a pandemic-era innovation into a robust tool for AMR surveillance. Aligning advances in sewage monitoring with ethical frameworks, policy integration, and equitable infrastructure development offers a path to detect and mitigate emerging resistance before the next crisis. Here, emerging resistance includes novel ARGs not yet reported clinically, the transfer of known ARGs into new pathogenic hosts, and their introduction into previously unaffected regions. With the scientific evidence and technical capacity already available, the next step is to integrate WBS within international AMR strategies and long-term response planning.
